# Executive Performance Is Associated With Rest-Activity Rhythm in Nurses Working Rotating Shifts

**DOI:** 10.3389/fnins.2022.805039

**Published:** 2022-02-24

**Authors:** Xiuli Zhao, Quan Tang, Zhengzhi Feng, Hóngyi Zhào

**Affiliations:** ^1^Department of Neurology, NO 984 Hospital of PLA, Beijing, China; ^2^Department of Medical Psychology, NO 984 Hospital of PLA, Beijing, China; ^3^Department of Medical Psychology, Army Medical University, Chongqing, China; ^4^Department of Psychiatry, NO 984 Hospital of PLA, Beijing, China

**Keywords:** shift work, nurse, rest-activity rhythm, non-parametric analysis, executive functions

## Abstract

**Purpose:**

Rest-activity rhythms (RAR) is one of the most fundamental biological patterns that influence basic physical and mental health, as well as working performance. Nowadays, with the utilization of actigraphy device in detecting and analyzing RAR objectively, evidence has confirmed that RAR could be interrupted by shift work. Nurses need to participate shift work in their daily routine. The aim of the present study is to identify the RAR of nurses working rotating shifts and find out the relationship between RAR and executive function.

**Methods:**

A total of 11 female nurses on day-shift (DS), 11 female nurses on rotating-shift (RS) were recruited. Demographic features, non-parametric variables of RAR as well as sleep variables according to actigraphy data, and a series of cognitive function evaluations during work time were collected.

**Results:**

The Student’s *t*-test comparison revealed that RS group nurses showed significant shorter total sleep time (TST), lower level of interdaily stability (IS) and relative amplitude (RA), as well as poorer executive performance compared with DS nurses. The linear regression analysis demonstrated that several aspects of executive performance such as choice reaction test (CRT) (reflecting attention and concentration) and trail making test (TMT) -B (reflecting cognitive flexibility) were associated with IS in RS group nurses.

**Conclusion:**

These results elucidated that RAR was disrupted for nurses working rotating shifts, and executive performance was associated with IS from day to day in nurses working rotating shifts.

## Introduction

1A person’s physiological functions change periodically. These changes are known as biological rhythms and are induced by signals from internal molecular mechanisms. In human beings, sleep-wake activity follows a diurnal pattern which is called the rest–activity rhythm (RAR) (Li et al., 2021). In recent years, a variety of methods recording several days of continuous accelerometer data could be used to detect RAR features (Smagula et al., 2019). These methods made investigators measure and quantify RAR characteristics objectively possible. Furthermore, several studies implied that RAR characteristics might be associated with physical and mental health (Slyepchenko et al., 2019; Kume et al., 2020).

Shift work involves working irregular or unusual hours different from a daytime work schedule (Wang et al., 2011). Nurses, participating shift work have jobs which require them to be active during hours that constitute normal rest time for most people. It has been concluded that shift work could cause circadian disruption and sleep loss, resulting in cognitive impairments and even accidents (Kecklund and Axelsson, 2016).

Rest-activity rhythms is considered as a type of rhythm pattern parallel, but not exactly equal to sleep-wake cycle (Calogiuri et al., 2013). As has been mentioned by Calogiuri et al., “*adding analysis of the rest-activity rhythm using rhythmometric procedures to traditional actigraphic studies could provide a deeper understanding of possible disorders of sleep in relation to the entrainment and vice versa.*” It is reported that shift work and the associated modifications in daily routines affect the nurses’ circadian rhythm (Kang et al., 2015). Though amount of studies have elucidate the relationship between shift work and decline in work efficiency for nurses (Narciso et al., 2016; Merchaoui et al., 2017), whether disrupted RAR of nurses of shift work impact on their executive performance during work is not clear.

In this study, we sought to identify the RAR of nurses working in rotating shifts by analyzing actigraphy data through the lens of non-parametric variables, and find out the relationship between RAR and executive function in nurses working in rotating shifts.

## Methods

### Participants

From June 1, 2020 to December 31, 2020, 11 female nurses on day-shift (DS), 11 female nurses on rotating-shift (RS) were recruited. Our study was approved by the Academic Ethics Committee of the Biological Sciences Division of NO 984 Hospital of PLA in Beijing, China. Informed consent was obtained from each participant in the study.

### Procedure

The nurses worked 2 × four slow, forward-rotating arranged RS (day shift-evening shift-night shift-off) or consecutive 8 days DS (08:00–16:00). Before beginning the actigraphic monitoring, demographic characteristics were collected. The exclusion criteria were: nurses who was pregnant or feeding baby younger than 3 years; in the habit of alcoholic drinking, strong tea or caffe; using of sedatives or hypnotic drugs; suffering from metabolic diseases, neurological diseases, renal diseases, or mental disorders.

### Wrist Actigraphy

According to the procedure, each participant was instructed to wear an ActiGraph GT3X+ device (ActiGraph, Pensacola, FL, United States) on their non-dominant wrist for 24 h per day (except when bathing or swimming) for 8 days. Considering that longer wearing of actigraphy duration will impact on the compliance of nurses, we selected 8-day protocol. And 8-day (encompassing 2 day shift+2 evening shift+2 night shift+ 2 off) is the minimum possible to observe with some clarity the RAR in people with unconventional routines.

**FIGURE 1:**
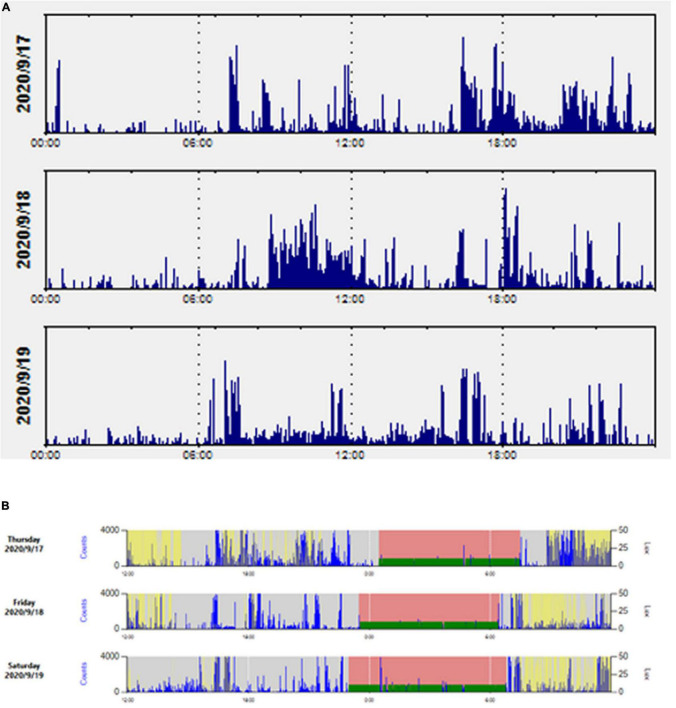
An example of a participant’s actogram, raw actigraphy data along with rest-activity rhythm **(A)** and sleep intervals **(B)** across the 24-h day.

At the end of the wear period, data were downloaded using ActiLife software (ActiGraph, Pensacola, FL, United States). All data files were visually screened for sufficient wear time and then processed for analysis (Figure 1).

### Data Processing

We calculated the non-parametric features using actigraph count data (vector magnitude counts) as follows: (1) the interdaily stability (IS); (2) the intradaily variability (IV); (3) the time occurrence and corresponding activity counts of the most active 10 h period (M10) and of the least active 5 h period (L5); and (4) the relative amplitude (RA) (Van Someren et al., 1999).

The IS, which provides information about the RAR synchronization to supposedly stable environmental stimuli, is calculated from the mean 24 h profile as the following formula 1:


$$I\,S=\frac{\text{n}\,\sum_{h=1}^{p}{\left({\bar{x}}_{h}-\bar{x}\right)}^{2}}{\text{p}\,\sum_{1}^{n}{\left(x_{i}-\bar{x}\right)}^{2}}$$

The IV, which provides information about the fragmentation in the RAR over the 24 h profile, calculated from hourly raw data as the following formula 2:


$$I\,V=\frac{\text{n}\,\sum_{i=2}^{p}{\left(x_{i}-x_{i-1}\right)}^{2}}{\left(n-1\right)\,\sum_{i=1}^{n}{\left(x_{i}-x\right)}^{2}}$$

In equation of IS or IV, n is the total number of data, p is the number of data entries per day, −*x*_*h*_ is the hourly average, −*x* is the average of all the data and−*xi* represents each hour of raw data. The IS ranges from 0 for Gaussian noise, which consisted of normal distribution noise, to 1 for a perfect synchronization. The IV measure ranges from 0 to 2, whose higher value indicates a more fragmented rhythm.

M10 is defined as the maximum sum of 10 consecutive hours of activity log. L5 is defined as the lowest sum of 5 consecutive hours of the activity log. RA is calculated as (M10-L5)/(M10+L5) (Witting et al., 1990).

### Objective Sleep Variables Detection

Sleep variables were also collected using a triaxial accelerometer (GT3X+; ActiGraph, LLC, Pensacola, FL, United States); this is a small (4.6 × 3.3 × 1.5 cm, 19 g), easy-to-wear device that offers the possibility of recording and scoring sleep–wake patterns during the night (Kim et al., 2015). Data from the Actigraphy data were downloaded and analyzed using the software with a 60 s epoch. Bedtime and wake time from the sleep diary were used to define rest intervals for calculation of sleep-wake variables. Actigraphy variables shown here were sleep latency (SL), sleep efficiency (SE), total time in bed (TTB), total sleep time (TST), wake after sleep onset (WASO), times of awakenings (TA) and average duration of awakenings (ADA).

### Cognitive Function Assessment

During the last DS, or night-shift work, similar to the procedure of Chang et al. (2011), all participants of each group completed a series of cognitive function tests, respectively. Considering that the main duties of nurses working night shifts at the study hospital include checking medical orders and prescriptions (Chang et al., 2011) correctly and quickly, the cognitive function assessments in the current study consisted of auditory verbal learning test-HuaShan version (AVLT-H), choice reaction test (CRT), digit symbol substitution test (DSST) and trail making test (TMT)-B (Zhào et al., 2019).

Auditory verbal learning test-HuaShan version is adapted by the Guo’s group (Yang et al., 2018) team based on the California Verbal Learning Test (CVLT) and Hong Kong auditory verbal learning test. This is a well-recognized measure for testing a person’s ability in different stages of working memory which includes encode, combine, store and recover verbal information (Zhang et al., 2019). Specifically, we informed the subjects to recall the words in advance. Then the neurologist read 12 Chinese words, including clothing, occupation, and flowers. We chose delayed memory mode, and the subject was asked to recall the words at the time point of 20 min after the words reading-out. CRT is a measurement of attention and concentration need to be completed on screen (Hou et al., 2020). During the test, stimulus (image of an arrowhead) was presented 40 times at random intervals on the screen. Participants were required to respond by pressing the button as quickly as possible with either hand. DSST is an evaluation of processing speed, derived from the battery of THINC-it (Hou et al., 2020). Participants are presented with six symbols, each representing one of six (1–6) digits. Subjects are asked to assign as many symbols as possible to the respective digits within 120 s. TMT-B is an assessment tool reflecting visual search speed and cognitive flexibility. It involves the participant connecting a trail through symbols (numbers 1, 2, 3……9 and corresponding Chinese characters 壹, 贰, 叁……玖) in the correct order (Wei et al., 2019). The time recorded began once the instruction to start was given and was stopped once the trail was completed.

### Statistical Analyses

The differences between the groups demographic data, as well as the differences between the groups on sleep variables, Non-parameter variables of RAR, CRT, DSST, and TMT-B performance were analyzed by Student’s *t*-test. A linear regression analysis was selected to find out the relationship between RAR (IS, IV, and RA), sleep variables (SL, SE, TTB, TST, WASO, TA, and ADA) and each domain of executive function. The data were expressed as the mean ± standard deviation, and a *P* value <0.05 was considered statistically significant. All statistical analyses were carried out under the statistical software package SPSS, 22.0 (IBM Corp., Armonk, NY, United States).

## Results

Nurses of both groups did not show significant difference in age, weight, height and body mass index (BMI). Most sleep variables except TST (357.34 ± 64.84 min vs. 414.59 ± 43.84 min, *P* = 0.025) did not reach statistical significance between RS group nurses and DS group nurses. On the contrary, non-parametric features of RAR such as IS (0.35 ± 0.10 vs. 0.59 ± 0.13, *P* = 0.000) and RA (0.80 ± 0.13 vs. 0.94 ± 0.02, *P* = 0.007), rather than IV (0.92 ± 0.18 vs. 0.91 ± 0.11, *P* = 0.933), differed statistically between group subjects. Details were shown in Table 1.

**TABLE 1:** Clinical and demographic characteristics of the nurses in different groups.

	Overall (*N* = 22)	RS (*N* = 11)	DS (*N* = 11)	*P* value
Age, years	26.27(3.60)	25.00(2.79)	27.55(3.98)	0.098
Weight, kg	58.23(9.44)	56.73(10.87)	59,73(8.00)	0.072
Height, cm	163.05(6.07)	165.36(7.84)	160.72(2.00)	0.470
BMI, kg/m^2^	21.88(3.27)	20.63(2.93)	23.13(3.21)	0.071
SL, minutes	5.48(3.77)	4.16(2.71)	6.79(4.31)	0.102
SE, %	83.80(6.33)	81.17(6.95)	86.43(4.55)	0.051
TTB, minutes	457.60(56.06)	437.51(54.84)	414.59(52.00)	0.093
TST, minutes	385.97(61.45)	357.35(64.84)	414.59(43.84)	0.025^#^
WASO, minutes	66.16(25.48)	76.01(26.19)	56.32(21.54)	0.068
TA, times	22.97(7.58)	23.54(6.68)	22.40(8.67)	0.732
ADA, minutes	3.03(1.59)	3.55(2.10)	2.51(0.52)	0.128
IS	0.46(0.16)	0.35(0.10)	0.56(0.13)	0.000^#^
RA	0.87(0.12)	0.80(0.14)	0.94(0.02)	0.007^#^
IV	0.91(0.15)	0.92(0.18)	0.91(0.11)	0.933
AVLT-H, scores	9.82(1.37)	9.90(1.30)	9.73(1.49)	0.764
CRT, seconds	0.36(0.06)	0.38(0.06)	0.33(0.05)	0.045^#^
DSST, scores	23.37(1.99)	23.73(1.95)	23.00(2.05)	0.404
TMT-B, seconds	19.09(3.26)	20.64(3.01)	17.55(2.84)	0.022^#^

*Mean (Standard Deviation). ^#^P < 0.05 RS relative to DS. BMI, body mass index; SL, sleep latency; SE, sleep efficiency; TTB, total time in bed; TST, total sleep time; WASO, wake after sleep onset; TA, times of awakenings; ADA, average duration of awakening; IS, interdaily stability; RA, relative amplitude; IV, intradaily variability; AVLT-H, auditory verbal learning test-HuaShan version; CRT, choice reaction time; DSST, digit symbol substitute test; TMT-B, trail making test-B.*

In addition, we evaluated the cognitive function of nurses in different groups. The RS group nurses spent significant longer time in completing CRT (0.38 ± 0.06 s vs. 0.32 ± 0.05 s, *P* = 0.045) and TMT (20.64 ± 3.01 vs. 17.55 ± 2.84, *P* = 0.022) relative to DS group nurses. Whereas, the scores on AVLT-H (9.72 ± 1.49 vs. 9.91 ± 1.30, *P* = 0.764) and DSST (23.00 ± 2.05 vs. 23.73 ± 1.95, *P* = 0.404) did not show obvious differences between groups. Details were shown in Table 1.

**FIGURE 2:**
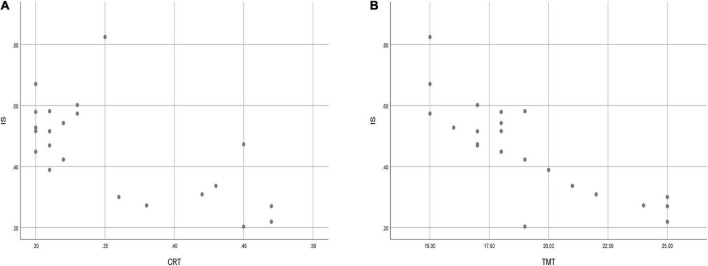
Scatter graphs demonstrating the relationship between IS and CRT **(A)**, as well as IS and TMT-B **(B)**.

Lastly, the association between cognitive function and RAR variables were detected using linear regression analysis in RS group nurses. The CRT time was negatively associated with IS (*P* = 0.031, standardized β = −2.690). TMT-B time was negatively associated with IS (*P* = 0.004, standardized β = −4.214). The scatter graphs demonstrating the relationship between IS and CRT, as well as IS and TMT-B, were shown in Figures 2A,B, respectively. Neither AVLT-H score nor DSST score was associated with RAR variables. However, cognitive function did not correlate with sleep variables in the present study. Details were shown in Table 2.

**TABLE 2:** Association between cognitive functional data and RAR non-parametric variables in RS group nurses.

	AVLT-H		CRT		DSST		TMT-B	
	Standardized β value	*P* value	Standardized β value	*P* value	Standardized β value	*P* value	Standardized β value	*P* value
SL, minutes	0.401	0.412	–0.161	0.739	0.358	0.576	0.536	0.355
SE, %	0.675	0.800	3.308	0.266	–0.499	0.889	2.834	0.387
TTB, minutes	–0.798	0.430	–1.451	0.195	–0.126	0.923	–1.233	0.311
WASO, minutes	–2.731	0.306	2.353	0.380	–0.273	0.934	–0.204	0.944
TA, times	2.134	0.120	0.369	0.756	–0.231	0.881	1.756	0.237
ADA, minutes	3.752	0.129	0.120	0.955	–0.203	0.849	2.954	0.268
IS	–1.529	0.170	–2.690	0.031^#^	–1.188	0.274	–4.214	0.004^#^
RA	0.661	0.530	0.077	0.766	0.910	0.393	2.067	–0.078
IV	–0.626	0.551	2.284	0.056	0.696	0.509	–0.669	0.525

*Data are standardized β values, except for P value. ^#^P < 0.05. SL, sleep latency; SE, sleep efficiency; TTB, total time in bed; TST, total sleep time; WASO, wake after sleep onset; TA, times of awakenings; ADA, average duration of awakening; IS, interdaily stability; RA, relative amplitude; IV, intradaily variability; AVLT-H, auditory verbal learning test-HuaShan version; CRT, choice reaction time; DSST, digit symbol substitute test; TMT-B, trail making test-B.*

## Discussion

In the present study, RS group nurses showed disrupted RAR and poorer sleep quality, reflected by lower IS and RA, as well as shorter TST in comparison with DS group nurses, respectively. In the view of RAR findings, as far as we know, this is the first study revealing the reduced stability of activity patterns from day to day and decreased amplitude of RAR in nurses working rotating shifts. In terms of sleep quality findings, our findings are in consistent with the systemic review reported by Chang and Peng (2021), showing that the sleep quality of nurses working rotating shifts was poorer than that of nurses working fixed day shifts. The only interesting point was that we only found statistical difference in TST between groups. However, unlike other studies, it seemed that rotation shift did not obviously change the levels of other sleep variables except TST in the current study. There are several explanations for this phenomenon: (1) compared with fixed night shift, rotation shift was an alternative reducing the amount of night work as possible (Khan et al., 2021); (2) it has been evidenced clockwise rotation of shift work could lead to better sleep quality relative to other methods (Barton and Folkard, 1993); and (3) sleep quality was evaluated using subjective methods such as Pittsburgh Sleep Quality Index in previous researches (Chang and Peng, 2021), while objective sleep quality measurement based on actigraphy data was utilized in our study. Considering that rotating shift workers are susceptible to mental disorders, particularly anxiety and depression (Kalmbach et al., 2015), it could be inferred that subjective sleep quality evaluation might overestimate the impact of shift work on sleep quality in nurses.

Apart from RAR and sleep variables, we also evaluate cognitive function of nurses in both groups during their working period. In detail, RS group nurses show deficits in executive function (attention and cognitive flexibility), with working memory and processing speed relatively spared. Previous findings of Özdemir and colleagues (Özdemir et al., 2013) implied that shift work could influence cognitive function in more domains. This discrepancy might be due to the study design, since we chose to collect cognitive function data during working period, instead of a fixed time 8:00 am (within working period for day shift nurses, but out working period for night shift nurses). Furthermore, linear regression analysis in RS group nurses demonstrated that poorer executive function was associated with less stable day-to-day RAR, instead of sleep quality. This finding is similar to the results derived from early Parkinson’s disease (Wu et al., 2018).

Interestingly, the impact of shift work on nurses seemed to differ according to the characteristics of the cognitive tests in the current study. This is different from other previously published studies demonstrating obvious declines in memory tests (Thun et al., 2021) and DSST (Zion and Shochat, 2018). This disaccord can be explained by the linear regression results. For example, Thun et al. (2021) reported that memory problems were associated with quick return and short sleep duration, and Zion and Shochat. (2018) found that DSST score was associated with clock time and sleepiness. All these parameters were relevant to fast rotating shift, instead of slow rotating shift. Actually, there existed evidence that unstable daily routine was associated with both memory and executive function in patients with liver cirrhosis (Kim et al., 2021) and Alzheimer’s disease (Alfini et al., 2021). The reason why we only find less aspects of cognition associated with IS might be that the participants recruited in the present study were all young healthy adults. As has been reported previously, cognitive declines began from as early as 30–40 years old and continued into older age (Ferguson et al., 2021). Thus, we inferred young adults might be more tolerant than elderly/patients toward IS disrupted condition.

Combined with the decorrelation between IS and sleep variables, all these results pointed out that RAR (e.g., IS, IV) is not merely a proxy for sleep in its association with cognition.

Several limitations of this study warrant consideration. First, the sample size was small. Second, we did not included nurses working fixed shifts, because rotating working shift is the most commonly found type of shift work for clinical departments in China. Third, we only recruited female nurses, in future study, we would collect male participants to overcome this shortcoming.

In summary, nurses working rotating shifts showed disrupted RAR, low sleep quality and impaired executive function. Poor executive function was associated with less stability of activity patterns from day to day.

## Data Availability Statement

The original contributions presented in the study are included in the article/supplementary material, further inquiries can be directed to the corresponding author.

## Ethics Statement

The studies involving human participants were reviewed and approved by the Ethics Committee of NO 984 Hospital of PLA. The patients/participants provided their written informed consent to participate in this study.

## Author Contributions

HZ and XZ were responsible for data collection. QT was responsible for manuscript writing. ZF was responsible for data analysis. HZ was responsible for the study design. All authors contributed to the article and approved the submitted version.

## Conflict of Interest

The authors declare that the research was conducted in the absence of any commercial or financial relationships that could be construed as a potential conflict of interest.

## Publisher’s Note

All claims expressed in this article are solely those of the authors and do not necessarily represent those of their affiliated organizations, or those of the publisher, the editors and the reviewers. Any product that may be evaluated in this article, or claim that may be made by its manufacturer, is not guaranteed or endorsed by the publisher.
